# In vitro analysis of the cytotoxicity and the antimicrobial effect of four endodontic sealers

**DOI:** 10.1186/1746-160X-7-15

**Published:** 2011-08-10

**Authors:** Ines Willershausen, Angelika Callaway, Benjamin Briseño, Brita Willershausen

**Affiliations:** 1 Department of Operative Dentistry, University Medical Centre of the Johannes Gutenberg University Mainz, Germany

**Keywords:** *in vitro *study, root canal sealer, *E. faecalis P. micra*, cytotoxicity

## Abstract

**Introduction:**

The aim of this study was to investigate *in vitro *the cytotoxicity and antibacterial properties of four different endodontic sealers using human periodontal ligament fibroblast cell proliferation and visual analysis of growth inhibition.

**Methods:**

A silicone (GuttaFlow), silicate (EndoSequence BC), zinc oxide eugenol (Pulp Canal Sealer EWT) and epoxy resin (AH Plus Jet) based sealer were incubated with PDL fibroblasts (10^4 ^cells/ml, n = 6) up to 96 h. Cell proliferation (RFU) was determined by means of the Alamar Blue assay. Cell growth and morphology was visualized by means of fluorescent dyes. Possible antibacterial properties of the different sealers were visualized by means of SEM (*Enterococcus faecalis; Parvimonas micra*).

**Results:**

Fibroblast proliferation depended on sealer and cultivation time. After 72 and 96 h GuttaFlow and EndoSequence BC showed relatively non-cytotoxic reactions, while Pulp Canal Sealer EWT and AH Plus Jet caused a significant decrease of cell proliferation (p < 0.001). Visualization of cell growth and morphology with various fluorescent dyes supplemented the results. No antibacterial effect of EndoSequence BC to *P. micra *was found, whereas GuttaFlow showed a weak, Pulp Canal Sealer EWT and AH Plus Jet extensive growth inhibition. Also, no antibacterial effect of GuttaFlow, EndoSequence BC or AH Plus Jet to *E. faecalis *could be detected.

**Conclusions:**

These *in vitro *findings reveal that GuttaFlow and EndoSequence BC can be considered as biocompatible sealing materials. However, prior to their clinical employment, studies regarding their sealing properties also need to be considered.

## Introduction

In recent decades, a considerable Improvement in endodontic methods, devices, and also in root canal filling materials, has occurred. Thus, patients as well as dental professionals are more inclined to favour tooth preservation over extraction of disputable teeth [1,2]. In consequence, since increased technical knowledge and scientific improvements have lead to higher treatment success rates, endodontic treatment and the subsequent restoration of the tooth should be considered as a therapy superior to implantation [3,4].

The choice of a biocompatible sealing material is crucial to the clinical success of endodontic therapy [5]. Although sealers were developed to be confined within the root canal system, their extrusion over the apical constriction is frequently observed [6,7]. Therefore, these materials should have good biocompatibility and be well tolerated by the peri-apical tissues [8]. The induction of a mild tissue reaction, together with cellular resorption of the sealing material in the case of extrusion over the apical foramen, needs to be evaluated. Several *in vitro, in vivo *and clinical studies [9-13] indicate that AH Plus, an epoxy resin-based root canal sealer, is suitable for successful endodontic therapy. This sealer remains popular despite its well-documented mutagenicity [14], cytotoxicity and the induction of a severe inflammatory response [15-17]. Besides cell dysfunctionality as a reaction to the epoxy resin-based sealing material [16], an intense inflammation characterized by the presence of lymphocytes, macrophages, giant foreign body cells as well as necrotic bone fragments in maxilla of guinea pigs after AH Plus implantation was observed. Due to its severe initial inflammatory reaction that diminished over time but persisted throughout the entire observation period, the authors [17] claim that this material does not possess enough biocompatible properties to be considered as an acceptable sealer for clinical use. Based on these contradictory results concerning an endodontic sealing material with a "gold standard" status [13], the tissue reaction induced by alternative sealers needs to be investigated in similar study designs to decide upon their potential clinical usage. GuttaFlow is a relatively new sealing material, which combines gutta-percha and sealer into an injectable system. According to the manufacturer, this system is based on polydimethylsiloxane with added gutta-percha and nano-silver particles (< 30 μm). Due to its viscosity, it is more likely to be extruded into the peri-apical tissue when placed under pressure [18]. However, it remains unclear which tissue reaction is caused by this material. In the study of AlAnezi *et al*. [19], the possible cytotoxicity of Endosequence BC Root Repair Material and grey and white MTA was evaluated. When exposed to these materials, the cells showed no significant difference in viability, while the cells in contact with AH 26 were significantly reduce in their viability.

Cleaning and shaping procedures are used to eliminate microorganisms from the root canal system during endodontic treatment. However, quite often a complete removal of bacteria is not possible [20]. In such cases it would be desirable that sealing materials have antimicrobial properties. Using either the agar diffusion test or the direct contact test or both, different endodontic sealers have already been assessed for a possible antibacterial effect, most often measured against strains of *E. faecalis *[21-28].

Baer and Maki [29] demonstrated that AH Plus and Pulp Canal Sealer EWT were not able to inhibit the growth of *E. faecalis*.

Therefore, the present *in vitro *study aimed at comparing the biocompatibility and the possible antibacterial effect on *E. faecalis *and *P. micra *of the four different root filling materials GuttaFlow, Endosequence BC, Pulp Canal Sealer EWT and AH Plus Jet.

## Materials and methods

### Sealing materials

For this *in vitro *study four different root canal sealers were chosen: GuttaFlow (Roeko, Coltène Langenau Germany, Batch No. 240412) consists of a polydimethylsiloxane matrix, is a cold flowable and self-curing sealer, which combines sealer and gutta-percha in one product; Endosequence BC Sealer (Brasseler, Savannah, GA, USA, Batch No. 0900458) is a premixed ready-to-use injectable material, based on a calcium silicate composition; Pulp Canal Sealer EWT (Pulp Canal Sealer EWT; SybronEndo, Orange, CA, USA, Batch No. 9-1222) is a zinc oxide eugenol based sealer; AH Plus Jet (Dentsply/Detrey, Konstanz, Germany, Batch No. 1004002041) is an epoxy resin based root canal sealer and consists of a paste-paste system, with paste A containing epoxy resin and iron oxide, and paste B containing amines and silicone oil.

**Figure 1:**
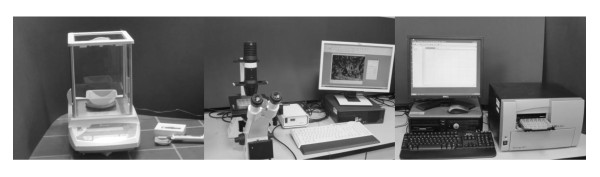
**Analytical balance (Pioneer PA64, Ohaus, Pine Brook, USA), left; inverted fluorescence microscope (Axiovert 40C/Carl Zeiss, Göttingen, Germany), middle; fluorescence/luminescence reader (Synergy HT-Reader, Biotek, Winooski, VT, USA), right**.

The sealers were prepared according to the manufacturers' recommendations. For the cell culture experiments, the materials (1.3 mg ± 0.1 mg) were placed at the junction between the base and wall of each multi-well cylinder (16 mm diameter; Greiner Bio-One, Frickenhausen, Germany), thus covering only a small area of the well. The amount of sealer was determined according to preliminary experiments and calculated by weighing the sealers with an analytical balance (Pioneer PA64, Ohaus, Pine Brook, USA, Figure 1, left). The sealing materials were allowed to set for 24 h.

To determine the bacterial colonization of root canal sealers, discs of equal size (Ø 12.5 ± 0.5 mm; thickness 2 ± 0.5 mm) were prepared under sterile conditions from the materials and allowed to set for 24 h.

### Cell culture

Human Periodontal Ligament Fibroblasts (Clonetics^® ^HPdLF Lonza, Switzerland) were cultured in Dulbecco's Modified Eagle Medium, supplemented with 10% foetal bovine serum, 2 mM L-Glutamine and 100U/100 μg/ml Penicillin/Streptomycin (Invitrogen, Paisley, UK), incubated at 37°C, in a humidified atmosphere containing 5% CO_2_, and a bidaily medium change. To assess the interaction of the sealing agents with the fibroblasts, different *in vitro *assays were carried out.

### Cell fluorescence

To demonstrate the interactions between cells and sealing materials, cells (20,000 cells/well) were stained with various fluorescent dyes and viewed with an inverted fluorescence microscope (Axiovert 40C/Carl Zeiss, Göttingen, Germany) at magnifications of × 25-400 (Figure 1, middle).

Phallacidin (BODIPY^® ^FL phallacidin; Invitrogen, Paisley, UK) selectively labels F-actin and was used to visualize the cytoskeleton. The blue-fluorescent DAPI nucleic acid stain (4',6-Diamidine-2'-phenylindole dihydrochloride; Roche Diagnostics, Mannheim, Germany) preferentially stains double stranded DNA. It yields highly fluorescent nuclei and no detectable cytoplasmic fluorescence. Blue fluorescence contrasts vividly with the green phallacidin staining.

Calcein-AM/Ethidium homodimer II staining (LIVE/DEAD^® ^Viability/Cytotoxicity Kit; Invitrogen, Paisley, UK), a two-colour fluorescence-based method, was used to measure the viability of the cultured cells, and to detect a possible cytotoxic effect of the sealers. Calcein AM is a fluorogenic esterase substrate that is hydrolysed intracellularly to a green fluorescent product, which is an indicator of live cells. Ethidium homodimer II enters cells through damaged membranes and intercalates with the DNA in the nucleus, emitting a red fluorescent signal.

### Cell viability assays

The four sealers were tested for possible effects on cell proliferation and metabolic activity of the PDL fibroblasts. Cell proliferation was quantitatively measured by means of the Alamar Blue assay (Alamar Blue Cell Viability Reagent; Biozol, Eching, Germany), which is based on detection of metabolic cell activity. The Alamar Blue reagent contains an indicator dye, which fluoresces in response to cell growth. The cells were incubated in a 96-well plate (10,000 cells/well) under standard conditions, and with 10% Alamar Blue for 96 h. At 0, 1, 6, 24, 48, 72, 96 h the fluorescence was measured at a wavelength of 560/20 and 620/40 nm with a fluorescence reader (Synergy HT-Reader, Biotek, Winooski, VT, USA). Cells without sealing material served as control. Logarithmic signals were converted to a linear scale and expressed as relative fluorescence units (RFU).

The cytotoxic potential of the four sealing materials was also investigated by means of the ToxiLight^® ^BioAssay Kit (Lonza Rockland, Rockland, ME, USA). This assay is a non-destructive, bioluminescent cytotoxicity assay, which quantitatively measures the release of Adenylate Kinase (AK) from damaged cells. The PDL fibroblasts were incubated under standard conditions in a 96-well plate (30,000 cells/well). After incubating the cells with the sealing agent for 24 h, the supernatants were mixed with AK detection agent. After 5 min incubation, the emitted light intensity is measured in a luminometer (Synergy HT-Reader, Biotek, Winooski, VT, USA, Figure 1, right). Logarithmic signals were converted to a linear scale and expressed as relative luminescence units (RLU).

### Bacterial colonization of root canal sealers

*Enterococcus faecalis *DSM 20478 was grown anaerobically for 24 h at 37°C in Schaedler broth (Becton Dickinson, Sparks, MD, USA). *Parvimonas micra *ATCC 33270 was grown anaerobically for 48 h at 37°C in Anaerobe Basal Broth (Oxoid, Basingstoke, Hampshire, England). Discs of equal size, prepared from the cements and set, were placed into Petri dishes, containing 25 ml of nutrient broth, inoculated with *E. faecalis *or *P. micra*, and incubated anaerobically at 37°C. After 24 h (*E. faecalis*) or 48 h (*P. micra*) of incubation, the discs were removed. To make the bacteria visible in a scanning electron microscope (SEM), the samples were fixed for 30 min in 3% formaldehyde at room temperature, and dehydrated by sequential washes through a series of 50 to 96% graded ethanol baths. After sputtering in a cold sputter unit, the samples were viewed in a DSM 962 SEM (Zeiss, Oberkochen, Germany) at an accelerating voltage of 10 kV.

### Statistical analysis

Six replicates per sealing material were used in the cell proliferation and cytotoxicity assays, and the results are presented as means ± standard deviation. The statistical analysis was performed using SPSS 15.0 (SPSS Inc., Chicago, IL) and SAS 9.2 (SAS Institute Inc., Cary, NC). The data were analysed by the Mann-Whitney-Test; p < 0.05 was chosen to define statistical significance, p < 0.01 was termed as highly significant.

## Results

**Figure 2:**
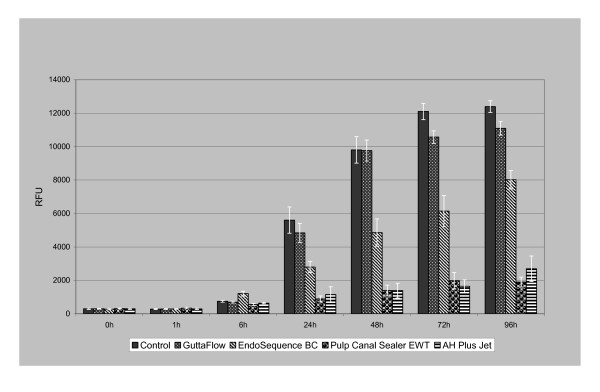
**Results of the Alamar Blue proliferation assay with PDL cells in contact with GuttaFlow, Endosequence BC, Pulp Canal Sealer EWT and AH Plus Jet, and with cells without root canal sealers (controls)**. After an incubation time of 96 h, the root canal sealers Pulp Canal Sealer EWT and AH Plus Jet significantly inhibited cell growth compared to GuttaFlow, Endosequence BC, and the control cells.

The Alamar Blue assay yields information about the proliferation rate of the PDL fibroblasts incubated with the different sealers over a period of 96 h. In this assay, high cellular proliferation rates were expressed as high relative fluorescence units (RFU). The here-investigated sealers influenced the proliferation and viability of the human periodontal ligament fibroblasts in different degrees (Figure 2). After an incubation time of 24 h, Pulp Canal Sealer EWT and AH Plus Jet significantly inhibited cell growth (p < 0.001). In contrast, incubation with GuttaFlow produced proliferation rates of the same order of magnitude as were found for the control group, and even promoted cell growth at 96 h. The proliferation rate of the cells in contact with Endosequence BC was significantly lower (p < 0.001) than of the controls, but significantly higher than cells in contact with Pulp Canal Sealer EWT and AH Plus Jet (p < 0.001).

**Figure 3:**
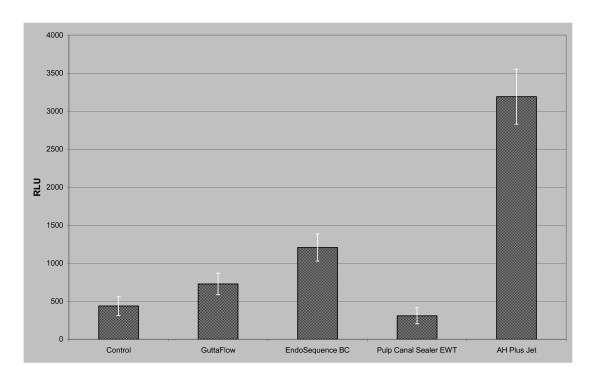
**Results of the ToxiLight^® ^BioAssay with PDL cells after 24 h**. The root canal sealer AH Plus Jet lead to a significantly higher release of Adenylate Kinase in comparison to the control cells and the other materials.

With the use of the ToxiLight^® ^BioAssay, it is possible to measure the quantitative release of Adenylate Kinase (AK) from damaged cells. High relative luminescence units (RLU) indicate a high release of Adenylate Kinase, which again is an indicator for damaged cells. The RLU are measured after the cells have been incubated with the respective sealing agents for 24 h. PDL fibroblasts without sealing material served as controls. Figure 3 shows the amounts of Adenylate Kinase released from the PDL fibroblasts incubated with the different sealing materials. Cells in contact with AH Plus Jet showed a significantly higher cytotoxicity than the controls and those incubated with the other sealing materials (p < 0.001).

**Figure 4:**
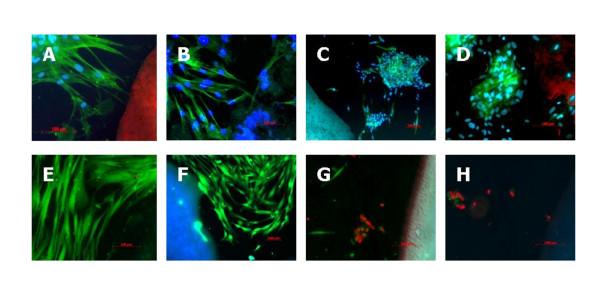
**The reaction of the PDL fibroblasts to GuttaFlow, Endosequence BC, Pulp Canal Sealer EWT and AH Plus Jet, stained with Phallacidin/DAPI (magnification A, B and D ×200, C ×100, bar = 100 μm) (A-D, upper panels) and with Calcein-AM/Ethidium homodimer II (magnification E, G and H ×200, F ×100, bar = 100 μm) (E-H, lower panels) is shown**. DAPI- stains the nucleus blue, and Phallacidin counterstains the cytoplasm green. The intact membrane of live cells is not permeable for Ethidium homodimer II.

The application of Phallacidin/DAPI was utilized to visualize nucleus and cytoplasm (Figure 4A-D). This revealed that the PDL fibroblasts in contact with the sealing materials were partially altered in shape, appearing round with no visible cytoplasmic structures. Hardly any cells are visible in close proximity to Pulp Canal Sealer EWT and AH Plus Jet (Figure 3C-D). Similar results were obtained when the cells were stained with Calcein-AM/Ethidium homodimer II (Figure 4E-H). Ethidium homodimer II enters into cells through damaged membranes, binding to nucleic acids, thereby producing a bright red fluorescence in dead cells. The intact cell membrane of live cells is not permeable for Ethidium homodimer II. In close proximity to Pulp Canal Sealer EWT and AH Plus Jet, most of the cells are damaged, as can be observed by the red colour in nearly all cells close to the sealers (Figure 4G-H).

### Bacterial growth

**Figure 5:**
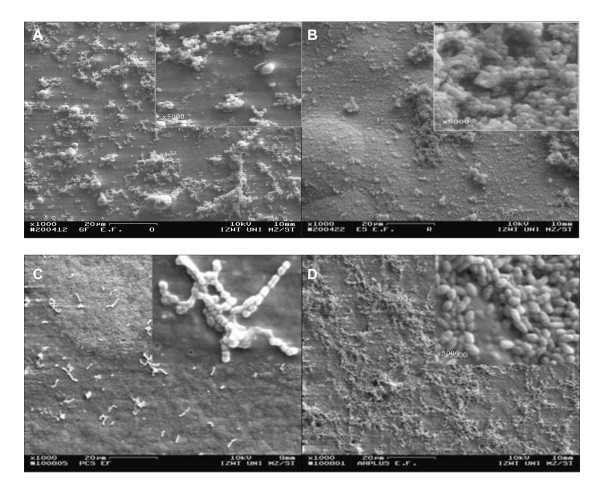
**Scanning electron micrographs of *E. faecalis *DSM 20478 grown on a disc prepared from GuttaFlow (A), EndoSequence BC (B), Pulp Canal Sealer EWT (C) or AH Plus Jet (D) after 24 h of incubation (A-D × 1000, insert × 5000, bar = 20 μm)**.

**Figure 6:**
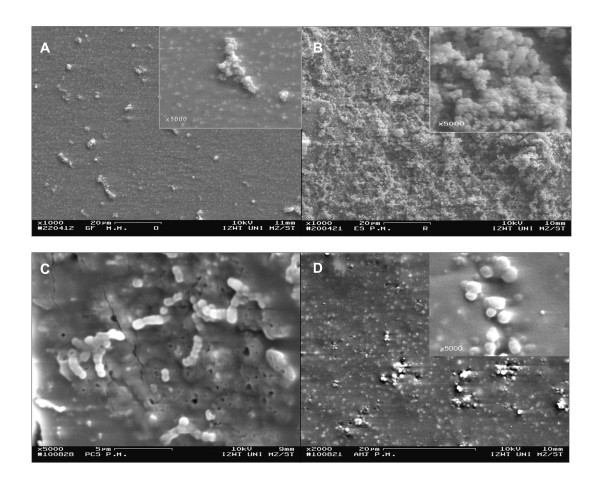
**Scanning electron micrographs of *P. micra *ATCC 33270 grown on a disc prepared from GuttaFlow (A), EndoSequence BC (B), Pulp Canal Sealer EWT (C) or AH Plus Jet (D) after 48 h of incubation (A-B: Magnification × 1000, insert × 5000, bar = 20 μm; C: Magnification × 5000, bar = 5 μm; D: Magnification × 2000, insert × 5000, bar = 20 μm)**.

No antibacterial effect of GuttaFlow, EndoSequence BC or AH Plus Jet to *E. faecalis *DSM 20478 could be detected by scanning electron microscopy. After 24 h of incubation, on GuttaFlow, EndoSequence BC and AH Plus Jet short chains, micro-colonies or layers of the bacteria, covering the complete surface, can be seen (Figure 5A-B, D). In contrast, Pulp Canal Sealer EWT is more sparsely colonized and only short chains of the cells can be detected (Figure 5D). The visual analysis of the scanning electron micrographs of the root canal sealers incubated for 48 h with *P. micra *ATCC 33270 shows on GuttaFlow only few bacteria organized in micro-colonies, whereas EndoSequence BC is uniformly colonized by the bacteria (Figure 6A-B). On Pulp Canal Sealer EWT and AH Plus Jet only at a magnification of 2000 or higher a few bacteria can be detected (Figure 6C-D).

## Discussion

The need for endodontic treatment is often associated with an inflammation caused by bacterial infection. For the successful root canal treatment, minimizing the possible inflammatory reaction caused by sealing materials, and suppressing bacterial growth are fundamental conditions. The goal of the endodontic treatment is to treat the teeth before a bacterial infection develops, and to use a biocompatible sealing agent. In case of an infection leading to pulp necrosis or of a bacterial contamination of the apical tissue, it is crucial for the outcome of the endodontic treatment to have a successful microbial elimination from the infected root canal system or to achieve a small enough number of microorganisms, which is clinically manageable [30]. It has also to be considered that in case of an inflammation caused by bacteria there will be a decrease of the pH in the peri-apical tissues, and thus there are special demands for the sealing agents [31]. The major task in reducing the bacterial load, concentrated in the apical region of the root canal, is achieved by the mechanical effects of instrumentation and the use of antimicrobial solutions for irrigation. Different studies have shown the essential role of chemo-mechanical procedures in eliminating the bacteria from the root canal system [32,33]. An antibacterial effect of root canal filling materials would be helpful, because if bacteria remain in dentinal tubules, this can serve as a reservoir for reinfection [34,35]. The bacteria chosen for this study were *E. faecalis *and *P. micra *(formerly *P. micros*). The former has been especially associated with endodontic failure, but has also been isolated from necrotic pulps. The latter organism, *P. micra*, has been isolated from asymptomatic and symptomatic primary endodontic infections, including abscesses as well as from endodontically treated teeth in need of re-treatment.

Calcium hydroxide is a well-described intra-canal material with an antibacterial effect, based on an alkaline pH, which has been demonstrated in several studies [36,37]. This substance was shown to inactivate bacterial lipopolysaccharides *in vivo *[38], but it is not effective in destroying all bacterial species associated with root canal infections.

Therefore, in this study the biocompatibility as well a possible antibacterial effects of four different types of root canal filling materials was tested; GuttaFlow, a gutta percha based material, the well described epoxy resin based AH Plus Jet, the Pulp Canal Sealer EWT as a zinc oxide eugenol based sealer, and the newly developed EndoSequence BC with a calcium silicate composition. An inflammatory reaction to various root canal filling materials is a frequent complication, and the knowledge of these characteristics is essential for the clinical success. Profound knowledge about the properties and responses to the used sealers is necessary to be better prepared for dealing with serious complications associated with over-extrusion of the material into the peri-apical area. The results of the cell proliferation assay showed that Pulp Canal Sealer EWT and AH Plus Jet significantly inhibited cell growth, and showed a lower biocompatibility in comparison to GuttaFlow and Endosequence BC. In the study of Brackett et al. [39], a severe and consistent cytotoxic response for Pulp Canal Sealer and AH Plus Jet was also observed, even over a time of up to 8 weeks, when tested in three different cell lines.

AH Plus also had a cytotoxic effect on human pulp cells *in vitro*, and showed other previously reported pro-inflammatory characteristics [40], The demands made on sealing materials have been modified in recent years. The primary requirement for sealing agents is to obturate the root canal system and to establish a hermetic seal of the apical area of the root. To achieve this is desirable to inhibit the growth of the microorganisms i.e. mainly bacteria remaining within the cleaned root canal system [41]. On the other hand, root canal sealers are required to demonstrate a good biocompatibility and are not supposed to irritate the peri-radicular tissue. The sealing ability of the root canal filling material should allow an adequate peri-apical healing after placement.

This is relevant, because the extrusion of sealing materials into the apical region with the direct contact to the peri-apical tissue is a well-described complication in endodontic treatment. The over-extrusion of non-resorbable materials or materials with slow breakdown is regarded as a critical factor in the apical healing process.

It is known that when certain non-resorbable materials, especially in the maxilla, are extruded into the human sinus, or are in contact with connective tissue, these materials are capable of triggering chronic inflammations [42,43]. The present findings with established root canal filling materials showed the challenging requirements for sealers. In addition, the paradoxical postulation of Grossmann is emphasized that root canal filling materials is supposed to inhibit the growth of all microorganisms, but at the same time show a good biocompatibility and not irritate the peri-radicular tissue.

## Conclusion

The present study shows that the materials Endosequence BC and GuttaFlow demonstrated a high biocompatibility, but had no antibacterial effect against *E. faecalis*. For *P. micra *a weak antimicrobial effect was observed with GuttaFlow. The sealers AH Plus Jet and Pulp Canal Sealer EWT showed a lower biocompatibility compared to Endosequence BC and GuttaFlow, but exerted a strong antimicrobial effect on *P. micra*.

## Competing interests

The authors declare that they have no competing interests.

## Authors' contributions

BW, IW and AC carried out the study. IW performed the statistical analysis. BW, AC, IW and BB conceived of the study, and participated in its design and coordination. All authors read and approved the final manuscript
